# 'Nasal mask’ in comparison with ‘nasal prongs’ or ‘rotation of nasal mask with nasal prongs’ reduce the incidence of nasal injury in preterm neonates supported on nasal continuous positive airway pressure (nCPAP): A randomized controlled trial

**DOI:** 10.1371/journal.pone.0211476

**Published:** 2019-01-31

**Authors:** Tanveer Bashir, Srinivas Murki, Sai Kiran, Venkat Kallem Reddy, Tejo Pratap Oleti

**Affiliations:** Department of Neonatology, Fernandez Hospital, Hyderguda, Hyderabad, India; Charité - Universitätsmedizin Berlin, GERMANY

## Abstract

**Background:**

With increasing use of nCPAP, the safety and comfort associated with nCPAP have come into the forefront. The reported incidence of nasal injuries associated with the use of nCPAP is 20% to 60%. A recent meta-analysis concluded that the use of nasal masks significantly decreases CPAP failure and the incidence of moderate to severe nasal injury and stress the need for a well powered RCT to confirm their findings.

**Methods:**

In this Open label, 3 arms, sequential, stratified randomized controlled trial, we evaluated the incidence and severity of nasal injury at removal of nCPAP when using two different nasal interfaces and in three groups (i.e. rotation group, mask continue group, prong continue group). Preterm infants with gestation ≤ 30 weeks and respiratory distress within the first 6 hours of birth and in need of CPAP were eligible for the study.

**Results:**

Among the 175 newborns included in the study, incidence of nasal injury in mask continue group [n = 19/57 (33.3%)] was significantly less as compared to prong continue group [n = 55/60 (91.6%)] and rotation group [33/ 58 (56.9%), p value <0.0001]. Median maximum nasal injury score was significantly less in Mask continue group as compared to Prong continue group and Rotation group [Injury Score 0 (IQR 0–1) vs. Injury Score 3 (IQR 2–5) vs. Injury Score 1 (IQR 0–2), p value = <0.0001] respectively. The proportion of infants failing nCPAP was similar across the three groups.

**Conclusion:**

nCPAP with nasal masks significantly reduces nasal injury in comparison with nasal prongs or rotation of nasal prongs and nasal masks. However, the type of interface did not affect the nCPAP failure rates.

## Introduction

Nasal CPAP is the standard respiratory therapy for preterm neonates with respiratory distress[1]. With increasing use of nCPAP, the safety and comfort associated with nCPAP have come into the forefront. The reported incidence of nasal injuries associated with the use of nCPAP is 20% to 60%[2–6] and range from simple blanching of the nasal tip to serious nasal septal necrosis and septal drop. Lower gestational age, lower birth weight and longer duration of nCPAP are important risk factors for nasal injury [3–5]. Apart from the known risk factors, type of nasal interface may also be an important determinant of nasal injury. A recent meta-analysis [7] compared nasal mask versus nasal prong for effectiveness and for nasal injury in preterm infants on CPAP, five trials were included, however effectiveness was evaluated in four trials (n = 459 infants) and moderate to severe injury was evaluated in three trials (n = 275 infants). Meta-analysis showed that nasal mask significantly decreased the risk of CPAP failure (4 RCTs [n = 459]; relative risk [RR]: 0.63; 95% confidence interval [CI]: 0.45–0.88; P = .007; I2 = 0%, NNT: 9), and the incidence of moderate to severe nasal trauma (3 RCTs [n = 275], RR: 0.41; 95%CI, 0.24–0.72; P = 0.002; I2 = 74%, NNT: 6). They concluded that the use of nasal mask significantly decreases CPAP failure and the incidence of moderate to severe nasal injury. The limitations of meta-analysis were small sample size, absence of a rotation group, heterogeneity of characteristics of participants and interventions and lack of standardized assessment of nasal injury. Since the site of injury is related to the pressure points of nasal interface and the pressure points of nasal mask are different from that of nasal prongs, there is need also to evaluate the role of rotation of nasal mask with nasal prongs for reduction of any nasal injury. In this study, we evaluated the incidence and severity of nasal injury at removal of nCPAP when using two different nasal interfaces and in three groups (i.e. rotation group, mask continue group, prong continue group).

## Materials and methods

The study was approved by our institutional ethics committee (12^th^ September 2016) and was registered in the clinical trial registry of India (CTRI/2017/04/008368, dated 19^th^ April 2017). The study recruitment started on 14^th^ September 2016 and follow up of all enrolled patients completed on 5^th^ June 2018. Consent was obtained from the parents for study enrolment and for publication of photographs of the infants (as outlined in PLOS consent form).

### Study design

Open label, 3 arms, sequential, stratified randomized controlled trial.

### Participant and study setting

Preterm infant with gestation ≤ 30 weeks and respiratory distress within the first 6 hours of birth and in need of CPAP were eligible for the study. Patients with perinatal depression (5-minute Apgar score of ≤3), major malformations and intubated at admission to the NICU were excluded.

#### Interventions

**Fig 1:**
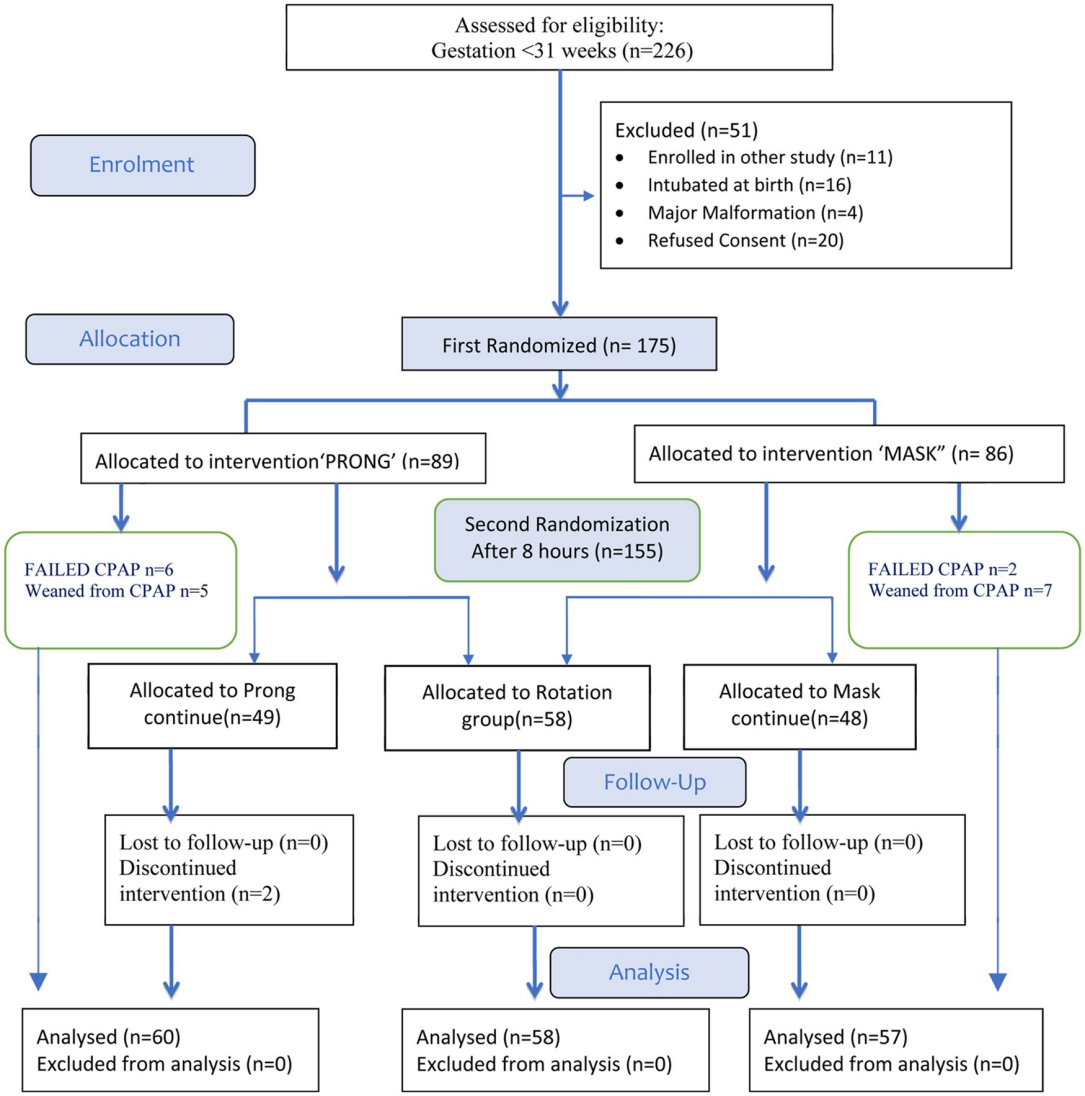
Consort flow diagram of the study participants.

Informed written consent was obtained from parents before birth or at admission to NICU. Neonates with onset of respiratory distress in the delivery room were transferred to the NICU on a T piece device (Neopuff). Randomization to nasal prong or nasal mask was done at admission to NICU. Randomization was stratified for gestation <28 weeks and 28 to 30 weeks. If two or more infants from a multiple pregnancy were eligible for enrolment, each of the infant was randomized separately. If the infant after the first randomization continued to be on the nCPAP after 8 hours of starting CPAP, a second randomization was done to allocate infant to same group assignment (prong continue, or mask continue) or to a rotation group (Fig 1). Each patient was eligible for 2 randomizations, one at enrolment (1:1 allocation for mask vs. prongs) and the other at 8 hours after study intervention (3:2 allocation, continue same interface vs. rotation group). The allocation was done by a web based random number sequence generator (www.randomization.com). Separate person not involved in the study generated the random sequence. Allocations were concealed by placing the allocation sequence in opaque, tamper proof, sealed, serially numbered envelopes. The nature of the intervention prevented us from blinding of the intervention from the investigators as well as the treating team. Attempts were made to minimize the bias by maintaining a strict study protocol.

#### CPAP nasal mask group

**Fig 2:**
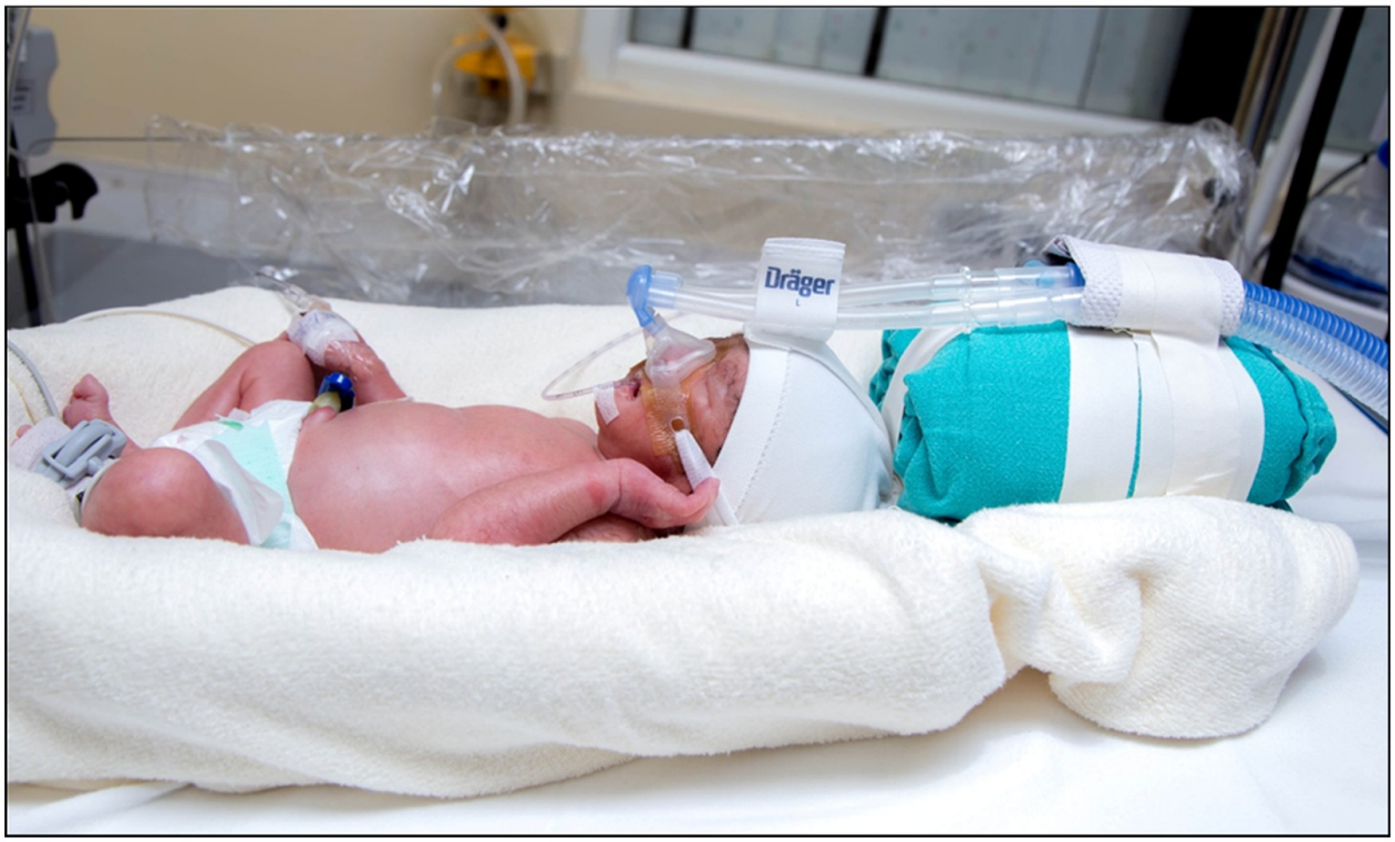
Drager BabyFlow mask.

The neonate was given CPAP support by using appropriately sized Drager BabyFlow mask (Fig 2) till CPAP was weaned off. The size of the mask chosen was as per the manufacturer’s instructions.

#### CPAP nasal prongs group

**Fig 3:**
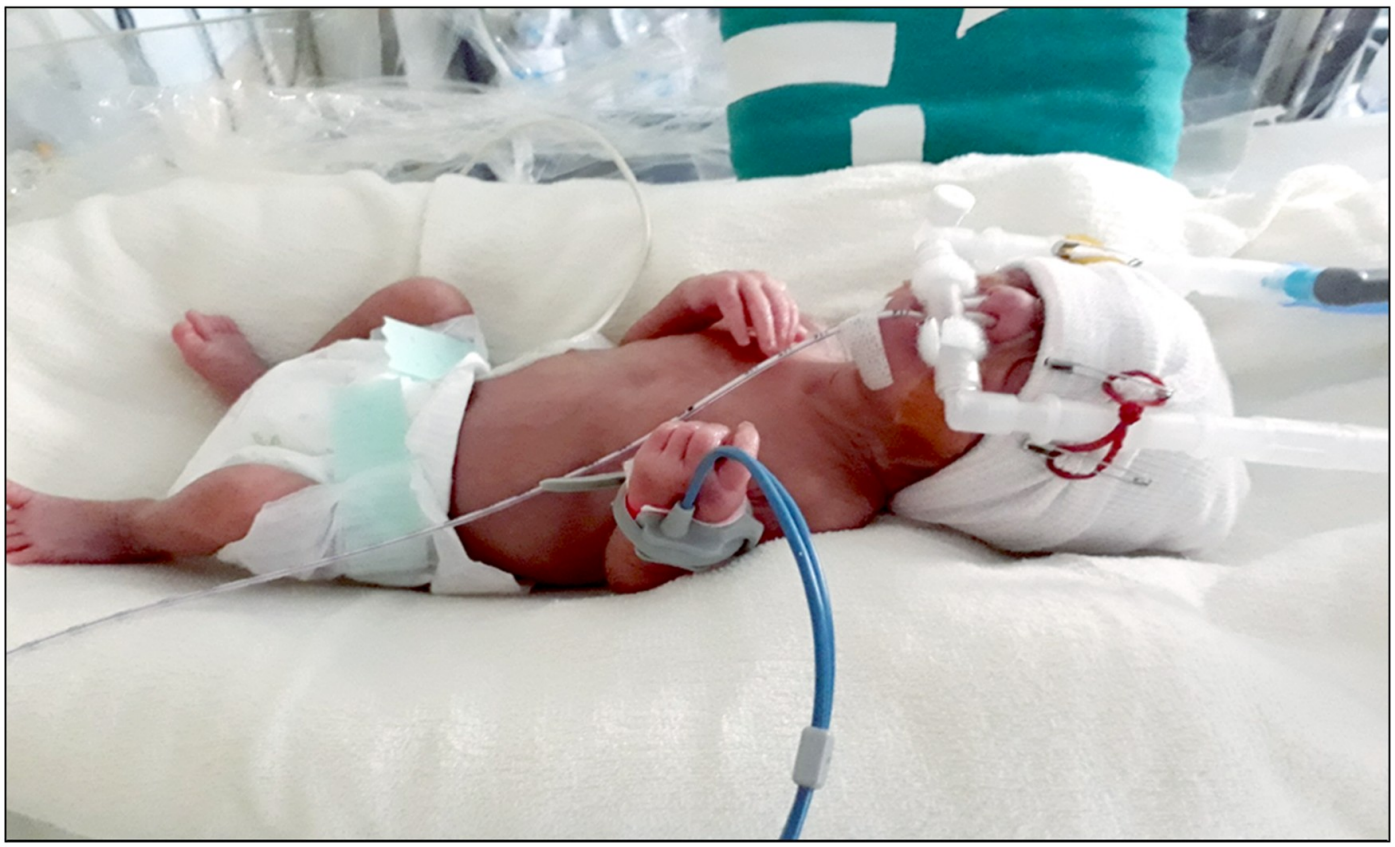
Hudson short binasal prongs.

The appropriately sized Hudson prongs (Fig 3) as per the manufacturer’s instructions was used for providing CPAP. The prongs were continued till CPAP was weaned off.

#### Rotating group (mask with prongs and vice versa)

The appropriately sized Drager BabyFlow mask and Hudson prongs were rotated every 8 hourly for delivering CPAP till CPAP was weaned off. Care was taken during these changes to minimize the duration of off-CPAP period.

**Fig 4:**
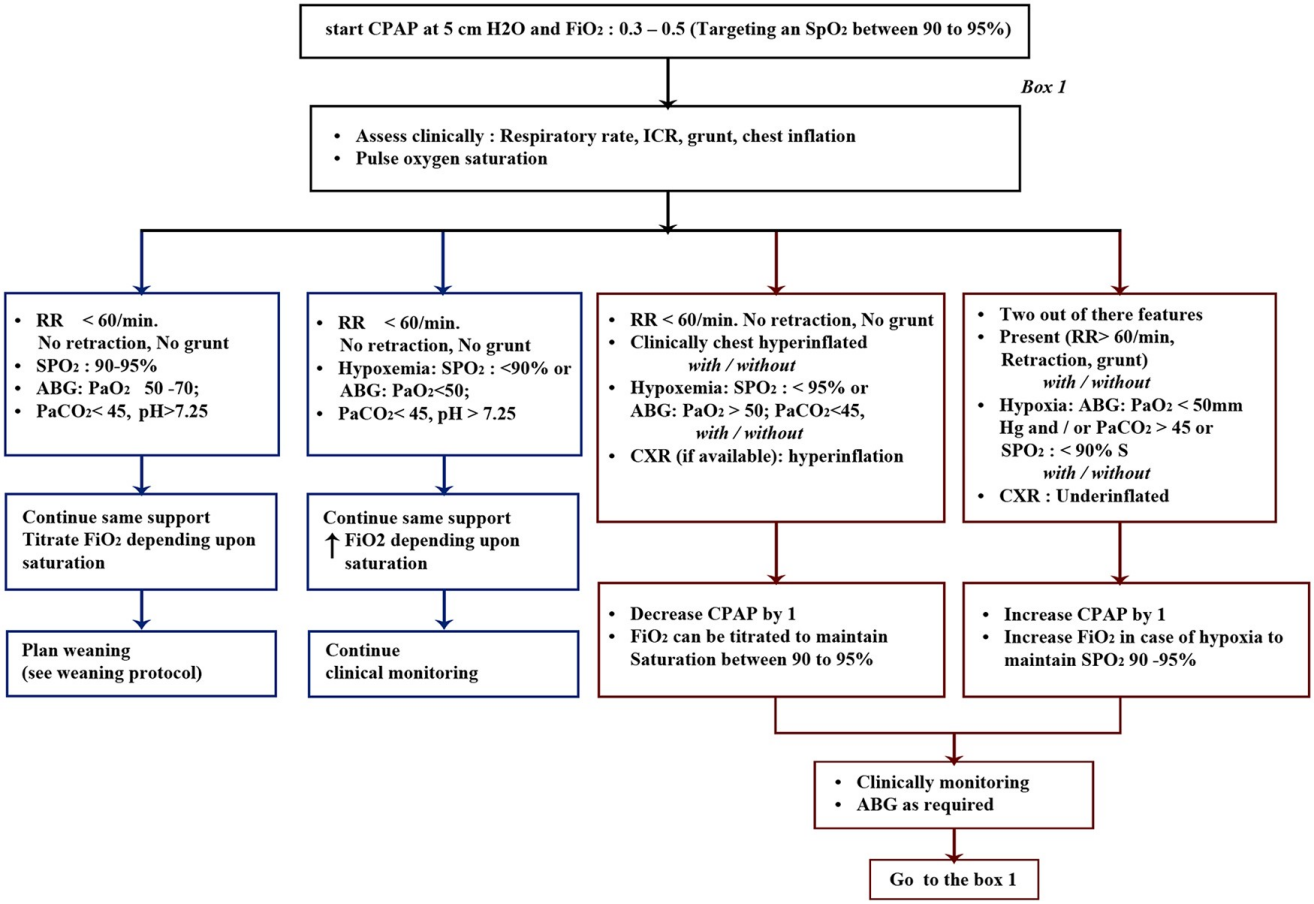
Clinical trial protocol for titration, weaning and removal of CPAP.

Skin barrier (DuoDERM Extra Thin Dressing) was applied at the pressure points in all infants as it was routine practice in the unit. Fisher and Paykel Bubble CPAP was used to deliver nCPAP in all infants. A protocol for titration, weaning and removal of CPAP was uniform in all the three groups (Fig 4). The gas delivered in both the devices was heated and humidified to attain a gas temperature of 37-degree C at the level of the nostrils. The humidifier used in both the groups had a flow-based servo-humidification control mechanism to ensure appropriate humidification.

nCPAP was removed if the infant was hemodynamically stable, had good respiratory efforts and had minimal or no recessions with SpO2 between 90 to 95% on minimal nCPAP of 4 cm of H_2_O and FiO_2_<30%.

All infants in this trial were weaned to high flow nasal cannula (HFNC) oxygen from nCPAP and if infant remained stable on HFNC for at least 6 hours’ infant was considered to have reached the primary outcome. If the infant did not tolerate HFNC within the 6-hour trial period, the infant was restarted on nCPAP with the same group allocation as before. CPAP failure was considered if an infant receiving maximum support (CPAP pressure> 7cm of H_2_O or FiO2 >0.60) had SpO2 <90% or had severe metabolic acidosis or respiratory acidosis (pH <7.20 and PaCO_2_>60 mm of Hg).

Surfactant and use of caffeine were as per the existing unit protocol and was similar among all the study infants. Monitoring for position of nasal prongs, prong displacements, delivered nCPAP pressures and adequacy of nCPAP was done hourly and recorded in structured proforma by the bedside nurse.

### Outcomes

Six hours after the removal of nCPAP, infants were assessed for nasal injury (Table 1) and digital photographs were taken that were later reviewed by a senior neonatologist who was blinded to the study group allocation. The incidence and severity of nasal injury were the primary outcome of the study. Bedside subjective assessment of nasal injury was also done by one of research assistants and this included; a) dilation of nares, b) columella indentation or excoriation, c) notching on the bridge of the nose, d) altered shape of nose and e) redness /indentation/bleeding/excoriation of any area of the nose.

**Table 1:** Nasal injury assessment score chart[8,9].

**Tip of Nose**	0 = Normal 1 = Red 2 = Red + indent 3 = Red/indent/skin breakdown 4 = As above +tissue loss
**Nasal Septum**	0 = Normal 1 = Red 2 = Red + indent 3 = Red / indent / skin breakdown 4 = as above + tissue loss
**Nostrils**	0 = Normal 1 = Enlarged 2 = Enlarged and prong shape 3 = Red, bleeding 4 = As above + skin breakdown
**Nose Shape**	0 = Normal 1 = Pushed up/back but normal 2 = Pushed up and shortened. No normal orientation when prongs removed.
**Bridge of the nose**	0 = Normal 1 = Red 2 = Red + indent 3 = Red/indent/skin breakdown 4 = As above +tissue loss
**Upper lip**	0 = Normal 1 = Red 2 = Red + indent 3 = Red/indent/skin breakdown 4 = As above +tissue loss

**Nasal injury scoring**:

0 = No injury, 1–4 = mild injury, 5–6 = moderate injury, ≥7 = severe injury

The secondary outcomes of the study included nCPAP failure, duration of nCPAP, duration of oxygen, culture positive sepsis, BPD, Intraventricular haemorrhages (IVH) grade ≥ 3. cystic periventricular leukomalacia (PVL), retinopathy of prematurity and mortality. Data were collected until death or discharge from the hospital.

### Sample size

We assumed an incidence of nasal injury of 50% among the control group at the removal of CPAP from literature review and previous study from our center [2–6,9]. Assuming three group comparisons with a minimum effect size of 30% with 80% power, alpha error of 5% the sample size calculated was 48 in each group and assuming an attrition rate of 10% we needed 54 neonates in each group. The sample size was estimated by using Compare k Proportions: 1- way ANOVA Pairwise test (www.powerandsamplesize.com). We enrolled patients till a minimum of 54 infants were enrolled in each group.

### Statistics

Categorical outcome variables were analysed by Chi square test with continuity correction or Fisher’s exact test. Normal distributed independent variables were compared by ANOVA test, whereas a non-parametric test (Mann-Whitney U) was used for variables with a skewed distribution. An Intention to treat analysis was done (ITT). All analysis was done using IBM SPSS version 21 and Microsoft Excel. A P value of less than 0.05 was considered significant.

## Results

Among the 175 newborns included in the study, after the first randomization 89 infants were allocated to the prong group, of which 6 infants failed nCPAP and 5 infants were weaned off in the first 8 hours leaving 78 infants for second randomization, i.e. 49 to prong continue and 29 infants to rotation group. Similarly, after the first randomization 86 infants were allocated to the mask group, of which 2 infants failed nCPAP and 7 infants were weaned off in the first 8 hours leaving 77 infants for second randomization, i.e. 48 to mask continue and 29 infants to rotation group (Fig 1). Mean gestation and mean birth weight of the study population was 28.93±0.1.32 weeks and 1.106±0.23 kg respectively. Twenty-eight infants (16%) and one hundred and forty-seven infants (84%) had gestation <28 weeks and 28 to 30 weeks respectively. Sixty infants (34.3%) had a birth weight <1000 grams. One hundred and thirty-one infants(n = 131) had respiratory distress syndrome and of them one hundred and twelve infants (85.5%) received surfactant. The median age of starting CPAP was 20 minutes. Baseline antenatal and neonatal characteristics were similar among the three groups (Table 2).

**Table 2:** Baseline characteristics.

Category	Subcategory	Prong continue group(no = 60)	Mask continue group(no = 57)	Rotation group(no = 58)	p- Value
Gestation Median (IQR)		29(28–30)	30(29–30)	29(28–30)	0.481^a^
Birth weight (Kg)Mean ±SD		1.06 ± 0.234	1.14 ± 0.206	1.12 ± 0.239	0.104^b^
Male n (%)		37(61.7)	33(57.9)	32(55.2)	0.772^c^
SGA (<10^th^ Centile)n (%)		10(16.7)	6(10.5)	5(8.6)	0.371 ^c^
Singleton n (%)		43(71.7)	40(70.2)	38(65.5)	0.601 ^c^
Maternal Age (yrs) Mean ±SD		29.03 ± 4.403	30.35 ± 6.004	29.36 ± 4.432	0.337 ^b^
PPROM n (%)		16(26.7)	19(33.3)	18(31.0)	0.727 ^c^
PIH n (%)		21(35.0)	21(36.8)	14(24.1)	0.285 ^c^
Doppler compromise n (%)		14(23.3)	10(17.5)	8(13.8)	0.401^c^
Antenatal Steroids	Partial n(%)	14(23.3)	6(10.5)	16(27.6)	0.117 ^c^
	Complete n(%)	46(76.7)	49(86.0)	41(70.7)	
C-section n (%)		47(78)	47(82.5)	42(72.4)	0.429 ^c^
RDS n (%)		47(78.3)	39(68.4)	45(77.6)	0.393 ^c^
TTNB n (%)		13(21.7)	18(31.6)	13(22.4)	0.393 ^c^
Pneumonia n (%)		5(8.3)	7(12.3)	6(10.3)	0.78 ^c^
Age at randomization(minutes)Median (IQR)		20(15–20)	20(20–20)	20(15–20)	0.379 ^a^
nCPAP at start Median (IQR)		5(5–6)	5(5–5)	5(5–5)	0.374 ^a^
FIO2 at start Median (IQR)		30(30–40)	30(30–40)	30(30–40)	0.901 ^a^

SD—Standard deviation, IQR Inter quartile range.

Values represented as mean (SD), median (IQR) or numbers (proportion); analysis by

^b^. ANOVA test,

^a^. Mann-Whitney U test,

^c^. Chi square test or Fisher’s Exact Test.

Incidence of nasal injury in mask continue group [n = 19 (33.3%)] was significantly less as compared to prong continue group [n = 55 (91.6%)] and rotation group [33 (56.9%), p value <0.0001]. All grades of nasal injuries (mild, moderate and severe) were lower in the mask continue group compared to prong continue group and rotation group. Median maximum nasal injury score was significantly less in Mask continue group as compared to Prong continue group and Rotation group [Injury Score 0 (IQR 0–1) vs. Injury Score 3 (IQR 2–5) vs. Injury Score 1 (IQR 0–2), p value = <0.0001] respectively. Also, when comparing infants that underwent the second randomization, nasal injury was significantly lower in the mask continue group (22/49, 45% vs.0/48 vs. 3/58, 5.2%, p<0.0001) (Table 3).

**Table 3:** Primary nasal injury outcomes.

Category	Subcategory	Prong continue group (no. 60)	Mask continue group (no. 57)	Rotation group (no. 58)	p- Value
Nasal injury n (%)	Any	55(91.6)	19(33.3)	33(56.9)	<0.0001^c^
	Mild	33(55.0)	19(33.3)	30(51.7)	0.042 ^c^
	Moderate	14(23.3)	0(0.0)	3(5.2)	<0.0001^c^
	Severe	8(13.3)	0(0.0)	0(0.0)	<0.0001 ^c^
Moderate to severe nasal injury n (%)		22(36.7)	0(0.0)	3(5.2)	<0.0001 ^c^
Erythema n (%)		39(65)	19(33.3)	32(55.2)	0.002 ^c^
Dilation of nares n (%)		19(31.7)	0(0.0)	1(1.7)	<0.0001 ^c^
Columella indentation/excoriation n (%)		15(25)	0(0.0)	2(3.4)	<0.0001 ^c^
Notching on bridge of nose		0	0	0	
Altered shape of nose		13(21.7)	0(0.0)	3(5.2)	<0.0001 ^c^
indentation/excoriation/bleeding any other part of nose n (%)		11(18.3)	0(0.0)	0(0.0)	<0.0001 ^c^
Maximum Nasal Injury Score Median (IQR)		3(2–5)	0(0–1)	1(0–2)	<0.0001^a^

SD—Standard deviation, IQR Inter quartile range.

Values represented as mean (SD), median (IQR) or numbers (proportion);

Values represented as mean (SD), median (IQR) or numbers (proportion); analysis by

^a^. Mann-Whitney U test,

^c^. Chi square test or Fisher’s Exact Test.

The proportion of infants failing nCPAP in first 72 hours after birth was similar across the three groups. The duration of nCPAP in hours, duration of oxygen therapy in days and the proportion of infants with bronchopulmonary dysplasia (BPD) at 36 weeks were similar in Prong continue group, Mask continue group and Rotation group respectively. Eight infants in the Prong continue group (13.3%), four infants in the Mask continue group (7.0%) and one infant in the Rotation group (1.7%) died before discharge from the hospital (Table 4)

**Table 4:** Secondary outcomes.

Category	Subcategory	Prong continue group (no = 60)	Mask continue group (no = 57)	Rotation group (no = 58)	p-Value
CPAP Hours Median (IQR)		22.5(10.25–36)	20(10–36)	29.5(21.75–47.25)	0.054^a^
Oxygen (Days)Median (IQR)		5(3–18)	6(3–14)	6(4–18)	0.904 ^a^
CPAP failure rate n (%)		11(18.3)	13(22.8)	6(10.3)	0.198^c^
MV n (%)	Within 72 hours	11(18.3)	14(24.6)	5(8.6)	0.127 ^c^
	72 hours to 7 days	8(13.3)	3(5.3)	8(13.8)	
MV days(mean ± SD)		1.25 ± 2.95	1.46 ± 3.15	1.34 ± 4.35	0.952^b^
BPD (oxygen at 36 weeks) n (%)		3(5.0)	1(1.8)	1(1.7)	0.470 ^c^
Maximum nCPAP Median (IQR)		5(5–6)	5(5–6)	5(5–6)	0.744 ^a^
Surfactantn (%)		43(71.7)	31(54.4)	38(65.5)	0.144 ^c^
Age at surfactantMedian (IQR)		60(45–90)	60(45–112.5)	60(60–120)	0.591 ^a^
NEC Stage ≥IIA n (%)		4(6.7)	3(5.3)	8(13.8)	0.213 ^c^
Sepsis n (%)		19(31.7)	9(15.8)	16(27.6)	0.123 ^c^
IVH grade III or Parenchymal bleed n (%)		2(3.3)	1(1.8)	0(0.0)	0.378 ^c^
Cystic PVL n (%)		1(1.7)	0(0.0)	1(1.7)	0.613 ^c^
ROP with Laser n (%)		1(1.7)	1(1.8)	1(1.7)	0.999 ^c^
Hospital stay Median (IQR)		31 (16.75–45.75)	28(17.5–40.5)	31.5(21–51.5)	0.748 ^a^
Death n (%)		8(13.3)	4(7.0)	1(1.7)	0.067 ^c^

SD–Standard deviation, IQR Inter quartile range, MV—Mechanical Ventilation

Values represented as mean (SD), median (IQR) or numbers (proportion);

Values represented as mean (SD), median (IQR) or numbers (proportion); analysis by

^b^. ANOVA test,

^a^. Mann-Whitney U test,

^c^. Chi square test or Fisher’s Exact Test

On subgroup analysis of infants who underwent second randomization, CPAP failure [n = 5(10%) vs. n = 11(23%), vs. n = 6(10%); p = 0.11], median duration of CPAP hours [24hours, IQR 15–48 hours vs. 23hours, IQR 12.75–36 hours vs. 29.5 hours, IQR 21.75 to 47.25 hours, p = 0.21] and death [n = 5 (10%) vs. n = 3 (6.3%), vs. n = 1 (1.7%), p = 0.17], were similar in all the 3 groups.

## Discussion

In this trial comparing nasal prongs vs. nasal mask vs. rotation of nasal mask and nasal prongs as nasal interface for delivering nCPAP in preterm infants with respiratory distress, nasal mask is superior to nasal prongs and rotation of nasal mask with nasal prongs in reducing any nasal injury and moderate to severe nasal injury. In-fact, none of the infants in the nasal mask continue group had moderate or severe nasal injury. Ease of use, softness and design of the make of nasal mask may be the reasons for lesser nasal injury in this group. As the trial design mandated higher duration of nCPAP for the rotation group (as randomization occurred after 8 hours of CPAP) group, when comparing the three groups only for infants that underwent a second randomization, the incidence of moderate to severe nasal injury was lowest in the mask group.

In the recent meta-analysis [7] that compared nasal mask versus nasal prongs for effectiveness and for nasal injury in preterm infants on nCPAP, five trials were included and effectiveness was evaluated in four trials (n = 459 infants) and moderate to severe injury was evaluated in three trials (n = 275 infants). They concluded that the use of nasal masks significantly decreases CPAP failure [RR 0.63 (CI 0.45 to 0.88)] and the incidence of moderate to severe nasal injury [RR 0.41(CI 0.24 to 0.72)]. The proportion of infants with moderate to severe nasal injury in the meta-analysis was thirty-six out of one hundred thirty-seven (26%) versus fourteen out of one hundred thirty-eight (10%) in the prong group and mask group respectively. The limitations of the meta-analysis were small sample size, heterogeneity of characteristics of participants and interventions and lack of standardized assessment of nasal injury. Similar to the meta-analysis, in our trial the incidence of moderate to severe nasal injury was significantly lower in the mask group as compared to prong group. Our study has a large sample size with uniformity in patient enrollment, inclusion of patient with the highest risk of CPAP failure and nasal injury, use of Drager BabyFlow mask and Hudson prongs as intervention and a standardized and objective method of assessment and scoring of nasal injury.

In the only other trial by Newnam et al [10], that compared nasal mask with nasal prongs and rotation of nasal mask and prongs, the frequency and severity of nasal trauma in preterm VLBW neonates allocated to receive nCPAP (post-extubation) by either continuous nasal prongs (n = 21), continuous nasal mask (n = 35), or alternating mask/prongs (n = 22), skin injury was significantly less (erythema p < 0.001, excoriation p = 0.007) in the rotation interface group compared to the other two groups. Small sample size, enrolment of infants post-extubation, lack of sequential randomization, skewed sample size and birth weight distribution within the groups, use of subjective scale for nasal injury and assessment bias are the major limitation of this trial.

Unlike the results from the meta-analysis, the nCPAP failure rate in our study is similar across the three groups even when adjusted for the duration of nCPAP. However, similar to the meta-analysis [7] and other previous trials [3,11–13] proportion of infants with mortality, bronchopulmonary dysplasia, necrotizing enterocolitis, cystic PVL and culture positive sepsis remained the same between the groups in our study. The trend towards reduced deaths in the rotation group is a chance finding and needs to be explored further. Relatively larger sample size, stratified randomization, inclusion of infants at risk for nasal injury, uniform protocol, objective outcome criteria and complete follow up till discharge are the main strengths of this study. Inability to blind the intervention, sequential randomization at admission and at 8 hours (may have introduced a structural bias into the 3 study groups) are the main limitation of this study.

## Conclusion

nCPAP with nasal masks significantly reduces nasal injury in comparison with nasal prongs or rotation of nasal prongs and nasal masks. However, type of interface did not affect the nCPAP failure rates.

## Supporting information

S1 File Study protocol. (DOCX)

S2 File Consort checklist. (PDF)

S3 File Data excel file. (XLSX)
