# Supplementary material for: 'Nasal mask’ in comparison with ‘nasal prongs’ or ‘rotation of nasal mask with nasal prongs’ reduce the incidence of nasal injury in preterm neonates supported on nasal continuous positive airway pressure (nCPAP): A randomized controlled trial
Source: PLoS One. 2019 Jan 31;14(1):e0211476. doi: 10.1371/journal.pone.0211476 (PMC6355017; doi:10.1371/journal.pone.0211476)
Supplement: S1 File — (DOCX) [file pone.0211476.s001.docx]

**Title** :

**Can rotation of nasal interfaces decrease the incidence of nasal injury in neonates on nasal CPAP?: A Randomized controlled trial**

Name of Candidate : Dr. Tanveer Bashir

Mail ID : drtanveer.151@gmail.com

Subject : Neonatology

Hospital/Institute : Fernandez hospital, Hyderabad.

Guide : Dr.Srinivas Murki

Senior consultant, Dept. of Neonatology

Fernandez hospital

**Introduction**

Delivering Continuous Positive Airway Pressure (CPAP) has become the standard of respiratory care in neonates. Nasal CPAP remains an efficacious way of delivering CPAP in preterm neonates with respiratory distress^(1)^. With the increasing use of nasal CPAP, the safety and comfort associated with nasal CPAP has come into the forefront. The reported incidence of nasal injuries associated with use of CPAP is 20% to 60% ^(2-6)^ and range from simple blanching of the nasal tip to serious nasal septal necrosis and septal drop. These nasal injuries may lead to long-term consequences including cosmetic problems. Local pressure at the nasal interface compounded by the cutaneous vulnerability and certain anatomical peculiarities such as end-vascularization of the Columella and nostrils are the principle factors reported to be responsible for the nasal injuries^(7)^. Lower gestational age, lower birth weight and longer duration of CPAP and neonatal intensive care unit stay have been reported as risk factors for nasal injury^(3,6)^. Apart from these factors, type of nasal interface and CPAP delivery devices may be important determinants of nasal injury associated with nasal CPAP. In this study we plan to evaluate if rotation of nasal interface reduces the incidence of nasal injury in comparison to use of a single interface (prong or mask).

**REVIEW OF LITERATURE**

*Burden of Disease*:

Respiratory disorders are the commonest cause of neonatal morbidity requiring intensive care. The incidence of respiratory distress in preterm neonates varies from 4 to 7 per 100 live births. Respiratory Distress Syndrome (RDS) constitutes one third of cases of respiratory distress in preterm neonates.^(8)^ The risk of RDS is inversely proportional to the gestational age, with an incidence of about 50% in babies less than 30 weeks of gestational age.^(9)^ RDS is the commonest disorder requiring assisted ventilation in both developed and developing countries. Assisted ventilation and surfactant has been the standard of care for last two decades.^(10)^ Respiratory support practice in newborn intensive care continues to evolve rapidly. A host of new modalities and techniques have become available for the infant with respiratory insufficiency over the past decade.

*Historical Aspect and known facts*:

The first use of CPAP in neonates was reported by Gregory et al in 1971. It was given by a head chamber (Gregory box) and was instrumental in decreasing the mortality in RDS from 35 -55% to 15 -20%.^(11)^The primary role of the CPAP is to support the smaller airways and to prevent generalized atelectasis of surfactant deficient alveoli. This leads to improvement in the oxygenation and ventilation by opening of poorly ventilated air spaces, relieving local vasoconstriction and decreasing intrapulmonary right to left shunt. In the late 1970s and 1980s, infant ventilators were developed rapidly. The focus for respiratory care of the newborns shifted gradually towards mechanical ventilation, and the use of CPAP declined. Though invasive mechanical ventilation decreased the mortality, there was no considerable change in the incidence of bronchopulmonary dysplasia (BPD).

A study conducted by Avery et al in 1987 comparing the outcome in eight different academic centers in USA noted that the best survival without BPD was noted in institutions that used nasal CPAP extensively for the stabilization of very low birth weight (VLBW) babies.^(12)^ Schmölzer performed a meta-analysis and included 4 trials (COIN, SUPPORT, CURPAP and VON) with a total of 2780 preterm infants (<32 weeks GA). The pooled results for the combined outcome death or BPD showed a reduction in favour of delivery room NCPAP compared to intubation with a relative risk 0.90 (0.83-0.98 95% CI) and numbers needed to treat (NNT) of 25^(13)^. Currently nasal continuous positive airway pressure (NCPAP) is the most commonly used respiratory support for newborn infants. It is a technique for non-invasive respiratory support of both term and preterm infants with respiratory distress.

## Nasal Interface

The success of nCPAP is to a large extent dependent on the appropriate selection of a nasal interface. short binasal prongs, long nasopharyngeal prongs or nasal masks are the interfaces currently available to deliver the NCPAP. Meta-analysis by De Paoli AG et al ^(14)^ concluded that short binasal prongs are more effective than nasopharyngeal prongs. There are only few randomised controlled trials which have compared the nasal prong and nasal mask as interfaces for nasal CPAP in VLBW neonates with respiratory distress syndrome .

S-C Yong et al(2005)^(15)^Compared the incidence of nasal trauma associated with the use of prong or mask during nasal continuous positive airway pressure (nCPAP) support in very low birthweight (<1501 g) infants. They conducted a randomised controlled clinical trial at a tertiary care university hospital, Department of Paediatrics, Kuala Lumpur, Malaysia. All very low birthweight infants admitted to the neonatal intensive care unit between July 2001 and December 2003 who received nCPAP through the Infant Flow Driver were randomised to the use of either nasal prong or mask. The nasal cavity of these infants was inspected daily during the first week and then weekly until they were weaned off nCPAP. Of the 89 infants recruited, 41 were randomised to the mask group and 48 to the prong group. There was no significant difference in the incidence of nasal trauma between the two groups (p = 0.5). The primary site of trauma was at the junction between the nasal septum and the philtrum in infants in the mask group and the walls of the nasal septum in the prong group. Logistic regression analysis showed that duration of nCPAP was the only significant risk factor associated with development of nasal injury, after birth weight, gestational age, and nasal device used had been controlled for (adjusted odds ratio 1.04; 95% confidence interval 1.01 to 1.07; p = 0.003.

Emily A. Kieran et al 2012^(16)^ compared nasal mask vs nasal prong as an interface to deliver nCPAP with need for ventilation as primary outcome and nasal injury as secondary outcome.120 infants were enrolled. Thirty-two of 62 (52%) infants randomly assigned to prongs were intubated within 72 hours, compared with 16/58 (28%) of those randomly assigned to mask (P = .007). Nasal injury occurred in 2 infants in each group with no statistically significant difference. Authors concluded that in premature infants, NCPAP was more effective at preventing intubation and ventilation within 72 hours of starting therapy when given via nasal masks compared with nasal prongs with no increased risk of nasal injury.

Katherine M. Newnam et al 2014^(17)^A three group prospective randomized experimental design was conducted to identify differences in frequency and severity of nasal injuries when comparing various interfaces used during continuous positive airway pressure (CPAP) and identified risk factors associated with injury. Seventy-eight neonates <1500 gm were randomized into three groups: continuous nasal prongs; continuous nasal mask; or alternating mask/ prongs. Participants were block stratified according to BW into four categories: <750 g; 750–1000 g; 1001–1250 g; and 1251–1500 g. Known differences in skin integrity have been demonstrated with the lowest BW infants considered the most vulnerable; thus, stratification was used to keep the groups more homogeneous since it was expected that the <750 g group would contain the fewest patients. All infants were managed with the same type of nasal CPAP delivery system, the Cardinal™ variable flow driver with Air Life™ prongs/mask. Infants transported from the delivery room or outlying hospitals initially treated with nasal CPAP were also eligible for enrolment. Infants that were extubated to other respiratory support devices (high flow nasal cannula, vapor-therm or nasal cannula) based on clinical decision were excluded from enrolment unless nasal CPAP was indicated at a later time. Randomization was conducted by the respiratory therapist at the time of extubation and was accomplished using serially numbered opaque sealed envelopes which were colour coded according to predetermined BW categories (<750 g; 750–1000 g; 1001–1250 g; and 1251–1500 g) supporting block stratification. To determine nasal interface group assignment, the next sequentially numbered envelop was drawn according to the participant's BW and the appropriate nasal CPAP interface was placed on the infant to provide therapy. Repeated measures ANOVA with Bonferroni correction demonstrated that significantly less skin injury was detected in the rotation interface group when compared to both mask and prong groups. In the final stepwise regression model (F = 11.51; R^2^= 0.221; p = 0.006) significant predictors of skin injury included number of days on nasal CPAP (p < 0.001) and current mean post menstrual age (p = 0. 006). Reduced nasal injury was demonstrated using rotating mask/prong nasal interfaces.

Sorabh Goel, Jayashree Mondkar et al 2015 ^(18)^ compared the effectiveness of nasal continuous positive airway pressure delivered by Nasal mask vs Nasal prongs with nasal trauma as secondary outcome. They conducted a randomized controlled, open label, trial at tertiary care level III neonatal unit.118 preterm infants-gestational age (27-34 weeks) requiring nasal continuous positive airway pressure as a primary mode for respiratory distress were included . 61 received nCPAP with nasal Mask as interface and 57 received nCPAP with nasal Prong as interface. Nasal continuous positive airway pressure failure occurred in 8 (13%) of Mask group and 14 (25%) of Prongs group but was statistically not significant (RR 0.53, 95% CI 0.24-1.17) (P = 0.15). The rate of pulmonary interstitial emphysema was significantly less in the Mask group (4.9% vs. 17.5%; RR 0.28, 95% CI 0.08-0.96; P = 0.03). Incidence of moderate nasal trauma (6.5% vs 21%) (P=0.03) and overall nasal trauma (36% vs 58%) (P=0.02) were significantly lower in mask group than in the prongs group.

Aparna Chandrasekaran et al ^(19)^ compared nasal mask vs nasal prong as an interface to deliver nCPAP with FiO2 requirement at 6, 12 and 24 h of CPAP initiation as primary outcome and nasal injury as secondary outcome. they conducted a randomized controlled trial at teaching hospital in new delhi from April 2012 to June 2013. 72 neonates were enrolled in the study —37 were assigned to the nasal mask group and 35 to the nasal prongs group. They found that FiO2 requirement comparable between two groups. The incidence of any grade of nasal trauma during the first 72 h was comparable between the groups (RR 1.07, 95% CI 0.84–1.35, p = .59). The incidence of severe—grade II/III— nasal trauma was, however, significantly lower in the mask group (0 vs. 31%; p < .001). authors concluded that Nasal masks appear to be as efficacious as binasal prongs in providing CPAP with Masks being associated with lower risk of severe nasal trauma.

From The above review of literature it seems both short binasal prongs and nasal mask are equally effective in delivering nCPAP but the measure concern is nasal injury that varies from mild to severe enough requiring corrective surgery, so we are planning to compare whether rotation of nasal interfaces will decrease the incidence of nasal injury as compared to nasal mask or nasal prong alone in neonates on nasal CPAP.

**AIMS & OBJECTIVES**

AIM

To know whether rotation of nasal interfaces will decrease the incidence of nasal injury as compared to nasal mask or nasal prong alone in neonates on nasal CPAP.

OBJECTIVES

**Primary objective**

1. To identify the incidence and severity of nasal injury at removal of CPAP in all the three groups (i.e. rotation group, mask continue group, prong continue group) based on the objective scoring and a subjective assessment.

**Secondary objectives**

To compare the following among the groups (prongs vs. masks vs. rotation of mask and prongs)

- The need for mechanical ventilation.
- The duration of CPAP, oxygen and ventilation
- Incidence of bronchopulmonary dysplasia as defined by requirement of oxygen or any form of respiratory support till 28 days of life (BPD)
- Ventilator associated pneumonia (VAP) as per NEO-KISS criteria
- Intraventricular haemorrhages (IVH) as defined by Papille et al.
- Periventricular leucomalacia (PVL) at discharge according to de Vries criteria
- Mortality among the groups will be noted till the baby is in NICU

**MATERIAL & METHODS**

Study site:

NICU, Fernandez Hospital, Hyderabad.

Study design:

A Prospective, Randomized, Comparative study.

Sample size with justification:

Assuming an incidence of nasal injury of 50% among the control group at the removal of CPAP from literature review and previous study from our center^(2-6)^. Assuming three group comparisons with an effect size of 30% with 80% power, alpha error of 5% the sample size calculated is 48 in each group and assuming an attrition rate of 10% we need 55 neonates in each group. Sample size was estimated by using Compare k Proportions: 1- way ANOVA Pairwise test ([www.powerandsamplesize.com](http://www.powerandsamplesize.com)). We will enroll patients till a minimum of 55 infants are enrolled in each group.

Time frame:

From september 2016 to march 2018.

Study population

Neonates admitted in NICU for respiratory distress

Inclusion criteria

- All inborn neonates <=30 weeks of gestation and
- Developed respiratory distress (presence of at least two out of three signs: tachypnea > 60/min, intercostal retractions, and expiratory grunt) within 6 hours of life.

Exclusion criteria

- Patients who have Perinatal depression (5 minute Apgar score of ≤3),
- Patients with Life threatening congenital malformations such as congenital diaphragmatic hernia, and trachea-esophageal fistula, other malformations that would prevent from establishing CPAP due to the anatomical defect such as Pierre-Robinson syndrome and choanal atresia
- Conditions where CPAP would be contraindicated such as neonates with poor spontaneous respiratory efforts or apnea, worsening shock, suspected or proven persistent pulmonary hypertension of newborn, severe metabolic acidosis (pH<7.2 with base deficit > -10), severe respiratory acidosis (pH<7.2 with PaCO2>55mmHg) and massive pulmonary hemorrhage

**Methodology**

Enrolment and randomization process:

Eligible pregnant mothers at a gestational period of <=30weeks will be approached for an informed, written consent for participation in the study. Eligible infants will be stratified as per gestational age, viz. <28 weeks and 28^0/7^-30^6/7^weeks. Those who satisfied the inclusion and exclusion criteria after admission to the neonatal unit will be enrolled in the trial and will be randomly allocated to either mask or prong group.

A second randomization would be done to continue same interface or allocate to rotating group if the infant continues to be on the nasal CPAP after 8 hours of starting CPAP. Those randomized to rotating group will be subjected to mask interface if on prongs and those on prongs will be subjected to mask and the other group would continue with the same interface as before (Figure). Allocation will be done by a web based random number sequence generator ([www.randomization.com](http://www.randomization.com)). Separate person who is not involved in the study will generate the random sequence. Randomization will be stratified for gestation <28 weeks and 28 to 30 weeks. Allocations will be concealed by placing the allocation sequence in opaque, tamper proof, sealed, serially numbered envelopes. The nature of the intervention will prevent us from blinding of the intervention from the investigators as well as the treating team. However, the statistician and the neonatologist reviewing nasal injury images will be blinded from the study allocation details. Further attempts will be made to minimize the bias by maintaining a strict study protocol.

Gestational age will be calculated primarily based on mothers last menstrual period (LMP). In cases where LMP is unreliable, either an early dating scan or clinical postnatal assessment by new ballard score will be used to calculate the gestational age. Baseline demographic and clinical details such as antenatal steroids, evidence of chorioamnionitis, age at first surfactant, and clinical or echocardiographic evidence of patent ductus arteriosus, severity of illness and severity of respiratory distress [Silverman-Anderson score ^(20)^] will be recorded in a structured case record form. Neonates with onset of respiratory distress in the delivery room will be transferred to the NICU on a T piece device (Neopuff). The duration from birth to onset of intervention and duration of use of T piece will be recorded. The study intervention will start once the study subject is admitted to the NICU.
Interventions**:**

- **CPAP Nasal Mask Group**: The neonate will be given CPAP support by using appropriate sized Drager mask till CPAP is weaned off. The size of the mask chosen will be as per the manufacturer’s instructions.
- **CPAP Nasal Prongs Group**: The appropriate sized Hudson prongs as per the manufacturer’s instructions will be used for providing CPAP. The prongs will be continued.
- **Rotating group (Mask with Prongs and vice versa)**: The appropriate sized Drager mask and Hudson prongs will be rotated every 8 hourly (each nursing shift) for delivering CPAP. Care will be taken during these changes to minimize the duration of off-CPAP period.

Heating and humidification of gases: The gas delivered in both the devices was heated and humidified to attain a gas temperature of 37 degree C at the level of the nostrils. The humidifier used in both the groups had a flow-based servo-humidification control mechanism to ensure appropriate humidification. Protocol for titration, weaning and removal of CPAP is given in figure 1.

**OUTCOMES MEASURED**

Six hours after the removal of nCPAP, infants will be assessed for nasal injury (Table 1) and digital photographs will be taken that will be reviewed by a senior neonatologist blinded to the study group allocation. Incidence and severity of nasal injury is the primary outcome of the study. Bedside subjective assessment of nasal injury will also be done by one of research assistant and this included; a) dilation of nares, b) columella indentation or excoriation, c) notching on the bridge of nose, d) altered shape of nose and e) redness /indentation/bleeding/excoriation of any area of nose.

The secondary outcomes of the study includes nCPAP failure, duration of nCPAP, duration of oxygen, culture positive sepsis, BPD, Intraventricular haemorrhages (IVH) grade ≥ 3. cystic periventricular leukomalacia (PVL), retinopathy of prematurity and mortality. Data will be collected until death or discharge from the hospital.

**Figure 1:** Clinical trial protocol for titration, weaning and removal of CPAP


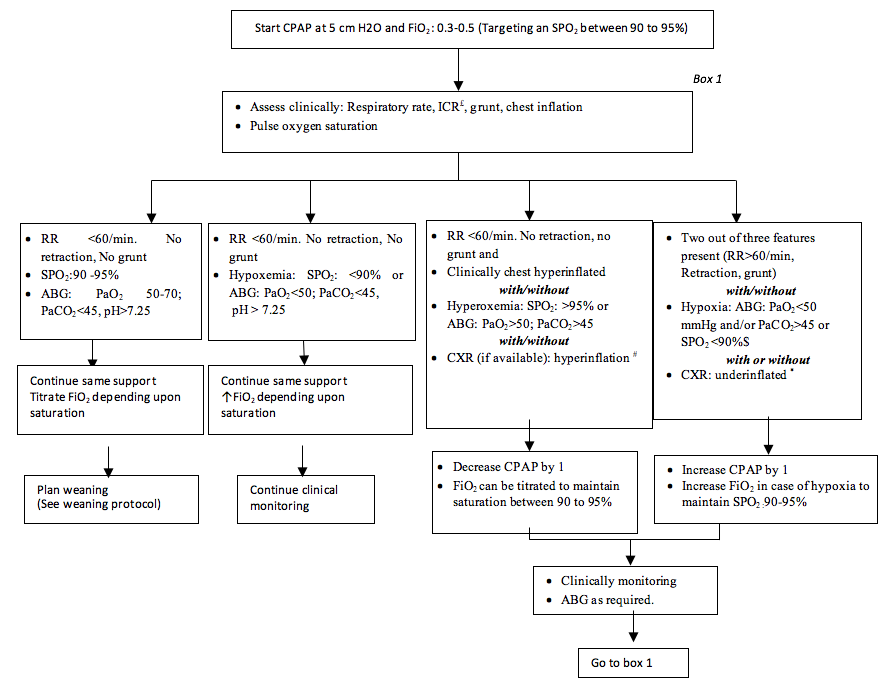


Note: Titration will be done primarily based on clinical features and pulse oxygen saturation; arterial blood gas and chest X ray will be used for decision making wherever available

* <6 and >8 posterior intercostals spaces is defined as under and over inflation respectively, ^£^ICR: Intercostal Retractions

**Weaning of CPAP**: Weaning from CPAP will be attempted if the infant will have good respiratory efforts and will be free of apneas and bradycardia in the past 24 hours and if one or more of the following will be present:

1. Stable or decreasing oxygen requirement over the past 12 hours
2. Chest wall hyperinflated* (either clinically or in a Chest X ray) with absence of retractions and a pulmonary air leak ruled out.

Note: If the infant did not tolerate the decrease in CPAP, as judged by an increase in oxygen requirement by 15% more than that in the preceding 12 hours, the CPAP pressure will be again increased by1 cm of H_2_O.

**Removal of CPAP/ EXIT from CPAP**:

CPAP will removed if following criteria are satisfied:

1. CPAP of 4 cm of H_2_O with FiO_2_<30%
2. Hemodynamically stable
3. Good respiratory efforts
4. Apnea and bradycardia free for last 24 hours

**Table 1 :**Nasal Injury Assessment score chart **^(21)^**

| **Tip of Nose** | 0=Normal  1= Red  2= Red + indent  3=Red/indent/skin breakdown  4=As above +tissue loss |
| --- | --- |
| **Nasal Septum** | 0=Normal  1=Red  2=Red + indent  3=Red / indent / skin breakdown  4=as above + tissue loss |
| **Nostrils** | 0=Normal  1= Enlarged  2= Enlarged and prong shape  3=Red, bleeding  4=As above + skin breakdown |
| **Nose Shape** | 0= Normal  1=Pushed up/back but normal  2=Pushed up and shortened. No normal orientation when prongs removed. |
| **Bridge of the nose** | 0=Normal  1= Red  2= Red + indent  3=Red/indent/skin breakdown  4=As above +tissue loss |
| **Upper lip** | 0=Normal  1= Red  2= Red + indent  3=Red/indent/skin breakdown  4=As above +tissue loss |

**Scoring:**

0= No injury

1-4= mild injury

5-6= moderate injury

≥7= severe injury

**Flow of study**

Intervention

Outcome

Screen for eligibility (n=)

Number of neonates enrolled

Initial Mask group

Initial Prongs group

Continuous Mask group

Interface

rotation

group

Continuous Prong

group

Interface

rotation

group

Number of neonates excluded =

Causes:

Primary and secondary outcomes

Enrollment

Randomization

**First Randomization**

**Second Randomization**

**Statistical analysis**

Categorical outcome variables will be analysed by Chi square test with continuity correction or Fisher’s exact test. Normal distributed independent variables will be compared by repeated measures ANOVA test whereas a non-parametric test (Mann-Whitney U) will be used for variables with a skewed distribution. An Intention to treat analysis will be done (ITT). All analysis will be done using IBM SPSS version 21 and Microsoft Excel. A P value of less than 0.05 will be considered significant.

**References**

1. De Paoli AG, Davis PG, Faber B, Morley CJ. Devices and pressure sources for administration of nasal continuous positive airway pressure (NCPAP) in preterm neonates. Cochrane Database Syst Rev 2008CD002977.
2. Robertson NJ, McCarthy LS, Hamilton PA, Moss AL. Nasal deformities resulting from flow driver continuous positive airway pressure. Arch Dis Child Fetal Neonatal Ed 1996;75:F209-12.
3. Yong SC, Chen SJ, Boo NY. Incidence of nasal trauma associated with nasal prong versus nasal mask during continuous positive airway pressure treatment in very low birthweight infants: a randomised control study. Arch Dis Child Fetal Neonatal Ed 2005;90:F480-3.
4. Buettiker V, Hug MI, Baenziger O, Meyer C, Frey B. Advantages and disadvantages of different nasal CPAP systems in newborns. Intensive Care Med 2004;30:926-30.
5. Rego MA, Martinez FE. Comparison of two nasal prongs for application of continuous positive airway pressure in neonates. Pediatr Crit Care Med 2002;3:239-43.
6. Fischer C, Bertelle V, Hohlfeld J, Forcada-Guex M, Stadelmann-Diaw C, Tolsa JF. Nasal trauma due to continuous positive airway pressure in neonates. Arch Dis Child Fetal Neonatal Ed 2010;95:F447-51.
7. Cartlidge P. The epidermal barrier. Semin Neonatol 2000;5:273-80.
8. Singh M, Deorari AK, Khajuria RC, Paul VK. A four year study on neonatal morbidity in a New Delhi hospital. Indian J Med Res 1991;94:186-92.
9. Rubaltelli FF, Bonafe L, Tangucci M, Spagnolo A, Dani C. Epidemiology of neonatal acute respiratory disorders. A multicenter study on incidence and fatality rates of neonatal acute respiratory disorders according to gestational age, maternal age, pregnancy complications and type of delivery. Italian Group of Neonatal Pneumology. Biol Neonate 1998; 74:7-15.
10. Welty S HT, Hansen TN, Corbet A. Respiratory distress in the preterm infant. *In*: Taeusch HW, Ballard RA, Gleason CA (*Eds*). Avery's Disease of Newborn, 8^th^ edn. Philadelphia, Saunders, 2005; pp 687-704
11. Dunn PM. Respiratory distress syndrome. Continuous positive airway pressure (CPAP) using the Gregory box. Proc R Soc Med 1974; 67:245-7.
12. Avery ME, Tooley WH, Keller JB, et al. Is chronic lung disease in low birth weight infants preventable? A survey of eight centers. Pediatrics 1987; 79:26-30.
13. Schmolzer GM, Kumar M, Pichler G, Aziz K, O'Reilly M, Cheung PY. Non-invasive versus invasive respiratory support in preterm infants at birth: systematic review and meta-analysis. BMJ 2013;347: f5980.
14. De Paoli AG, Davis PG, Faber B, Morley CJ. Devices and pressure sources for administration of nasal continuous positive airway pressure (NCPAP) in preterm neonates. Cochrane Database of Systematic Reviews 2008(1). accessed from https://www.ncbi.nlm.nih.gov/pubmed/18254011
15. [S-C Yong](http://fn.bmj.com/search?author1=S-C+Yong&sortspec=date&submit=Submit), [S-J Chen](http://fn.bmj.com/search?author1=S-J+Chen&sortspec=date&submit=Submit), [N-Y Boo](http://fn.bmj.com/search?author1=N-Y+Boo&sortspec=date&submit=Submit). Incidence of nasal trauma associated with nasal prong versus nasal mask during continuous positive airway pressure treatment in very low birthweight infants: a randomised control study. Arch Dis Child Fetal Neonatal 2005;90:F480-3.
16. [Kieran EA](https://www.ncbi.nlm.nih.gov/pubmed/?term=Kieran%20EA%5BAuthor%5D&cauthor=true&cauthor_uid=23090339), [Twomey AR](https://www.ncbi.nlm.nih.gov/pubmed/?term=Twomey%20AR%5BAuthor%5D&cauthor=true&cauthor_uid=23090339), [Molloy EJ](https://www.ncbi.nlm.nih.gov/pubmed/?term=Molloy%20EJ%5BAuthor%5D&cauthor=true&cauthor_uid=23090339), [Murphy JF](https://www.ncbi.nlm.nih.gov/pubmed/?term=Murphy%20JF%5BAuthor%5D&cauthor=true&cauthor_uid=23090339), O'Donnell CP. Randomized trial of prongs or mask for nasal continuous positive airway pressure in preterm infants. Pediatrics 2012;130:e1170-6.
17. Newnam KM, McGrath JM, Salyer J, Estes T, [Jallo N](http://www.ncbi.nlm.nih.gov/pubmed/?term=Jallo%20N%5BAuthor%5D&cauthor=true&cauthor_uid=25017108) and [Bass WT](http://www.ncbi.nlm.nih.gov/pubmed/?term=Bass%20WT%5BAuthor%5D&cauthor=true&cauthor_uid=25017108). A comparative effectiveness study of continuous positive airway pressure-related skin breakdown when using different nasal interfaces in the extremely low birth weight neonate. [Appl Nurs Res](http://www.ncbi.nlm.nih.gov/pubmed/25017108) 2015;28:36-41.
18. [Goel S](http://www.ncbi.nlm.nih.gov/pubmed/?term=Goel%20S%5BAuthor%5D&cauthor=true&cauthor_uid=26713987), [Mondkar J](http://www.ncbi.nlm.nih.gov/pubmed/?term=Mondkar%20J%5BAuthor%5D&cauthor=true&cauthor_uid=26713987), [Panchal H](http://www.ncbi.nlm.nih.gov/pubmed/?term=Panchal%20H%5BAuthor%5D&cauthor=true&cauthor_uid=26713987), [Hegde D](http://www.ncbi.nlm.nih.gov/pubmed/?term=Hegde%20D%5BAuthor%5D&cauthor=true&cauthor_uid=26713987), [Utture A](http://www.ncbi.nlm.nih.gov/pubmed/?term=Utture%20A%5BAuthor%5D&cauthor=true&cauthor_uid=26713987) and [Manerkar S](http://www.ncbi.nlm.nih.gov/pubmed/?term=Manerkar%20S%5BAuthor%5D&cauthor=true&cauthor_uid=26713987). Nasal Mask Versus Nasal Prongs for Delivering Nasal Continuous Positive Airway Pressure in Preterm Infants with Respiratory Distress: A Randomized Controlled Trial. [Indian Pediatr](http://www.ncbi.nlm.nih.gov/pubmed/?term=Sorabh+Goel%2C+Jayashree+Mondkar+et+al) 2015;52:1035-40.
19. Aparna Chandrasekaran, Anu Thukral, M Jeeva Sankar, Ramesh Agarwal, Vinod K Paul, Ashok K Deorari. Nasal masks or binasal prongs for delivering continuous positive airway pressure in preterm neonates—a randomised trial. Eur J Pediatr 2017;176:379-86
20. Silverman WA, Andersen DH. A controlled clinical trial of effects of water mist on obstructive respiratory signs, death rate and necropsy findings among premature infants. Pediatrics 1956;17:1-10.
21. Gupta S, Sinha SK, Tin W, Donn SM. A randomized controlled trial of post-extubation bubble continuous positive airway pressure versus Infant Flow Driver continuous positive airway pressure in preterm infants with respiratory distress syndrome. J Pediatr 2009;154:645-50.

**ANNEXURE - 1 :**

**STUDY PROFORMA**

**Title: Can rotation of nasal interfaces decrease the incidence of nasal injury in neonates on nasal CPAP?: A Randomized control trial**

**Screening Form**

| ***Purpose of the form:***   - *This form is meant for all preterm neonates with respiratory distress.* - *Collect the information from the patient file /attendant* | | | | | | | | | | | | | | | | | | | | | | | | | | | | |
| --- | --- | --- | --- | --- | --- | --- | --- | --- | --- | --- | --- | --- | --- | --- | --- | --- | --- | --- | --- | --- | --- | --- | --- | --- | --- | --- | --- | --- |
| **PART A: DETAILS OF THE INFANT (TO BE COLLECTED AT SCREENING)**   - **Please fill the following choices, unless otherwise specified** | | | | | | | | | | | | | | | | | | | | | | | | | | | | |
| S.No | Items | | Response | | | | | | | | | | | | | | | | | | | | | | | | | |
| 1 | Name of the Baby *(in block letters)* | B/o |  |  | |  | |  | | |  |  |  | |  | |  | |  |  |  | | |  | |  | |  |
| 2 | Mother’ Hospital registration number | |  | |  | | | |  | | |  | |  | | | |  | |  | | |  | | | |  | |
| 3 | Baby’s Hospital registration number | |  | |  | | | |  | | |  | |  | | | |  | |  | | |  | | | |  | |
| 4 | Date of birth ***(dd/mm/yy)*** | |  | | | |  | | | | |  | | | |  | | | |  | | | | |  | | | |
| 5 | Time of birth ***(24 hr format)*** | |  | | | | | | |  | | | | | |  | | | | | |  | | | | | | |
| 6 | Birth weight | |  | | | | | | |  | | | | | |  | | | | | |  | | | | | | |
| 7 | Length | | cm | | | | | | | | | | | | | | | | | | | | | | | | | |
| 8 | Sex | | Male/Female | | | | | | | | | | | | | | | | | | | | | | | | | |
| 9 | Head Circumference | | cm | | | | | | | | | | | | | | | | | | | | | | | | | |
| 10 | Resuscitation at Birth | | Y / N  Apgar (a) 1 min_____(b) 5 min______ | | | | | | | | | | | | | | | | | | | | | | | | | |
| 11 | Multiple births | | Single / Twin / Triplet | | | | | | | | | | | | | | | | | | | | | | | | | |
| **Inclusion Criteria** | | | | | | | | | | | | | | | | | | | | | | | | | | | | |
| S.No | Items | | Response | | | | | | | | | | | | | | | | | | | | | | | | | |
| 1 | Gestational age ≤ 30 weeks | | Y / N | | | | | | | | | | | | | | | | | | | | | | | | | |
| 2 | Respiratory distress within 6 hours of life | | Y / N | | | | | | | | | | | | | | | | | | | | | | | | | |
| 3 | Does the baby have all the inclusion criteria? | | Y / N | | | | | | | | | | | | | | | | | | | | | | | | | |
| **Exclusion Criteria** | | | | | | | | | | | | | | | | | | | | | | | | | | | | |
| S.No | Items | | Response | | | | | | | | | | | | | | | | | | | | | | | | | |
| 1 | Abnormalities of upper and lower airways precluding use of CPAP or HHHFNC (Pierre-Robin, Treacher Collins, Goldenhar, choanal atresia, cleft lip/palate) | | Y / N | | | | | | | | | | | | | | | | | | | | | | | | | |
| 2 | Serious abdominal, cardiac, or respiratory malformations including tracheal esophageal fistula, intestinal atresia, omphalocele, gastrochisis, or diaphragmatic hernia | | Y / N | | | | | | | | | | | | | | | | | | | | | | | | | |
| 3 | Required intubation at birth | | Y/N | | | | | | | | | | | | | | | | | | | | | | | | | |
| 4 | Perinatal depression (5 minute Apgar score of ≤3) | | Y/N | | | | | | | | | | | | | | | | | | | | | | | | | |
| **Consent**  ***(If response to all the exclusion criteria is No, obtain consent from parent/ attendant)*** | | | | | | | | | | | | | | | | | | | | | | | | | | | | |
| S.No | Items | | Response | | | | | | | | | | | | | | | | | | | | | | | | | |
| 1 | Written and verbal consent obtained from parent/ direct care taker of the baby? | | Y / N | | | | | | | | | | | | | | | | | | | | | | | | | |
| 2 | If ‘Y’, have you enrolled the baby? | | Y / N | | | | | | | | | | | | | | | | | | | | | | | | | |

**Enrolment Form**

| **Enrolment Number** |  |  |  |
| --- | --- | --- | --- |
| **Group** | **A or B or C** | | |

Group A -Prongs Group B- Mask Group C: Rotation group

| ***PURPOSE OF THE FORM:***   - *This form is meant to be filled only if patient satisfies all the criteria mentioned in screening form* - *Collect information from the file / patient attendant* | | | | | | | | | | | | | | | | | |
| --- | --- | --- | --- | --- | --- | --- | --- | --- | --- | --- | --- | --- | --- | --- | --- | --- | --- |
| **Part A 1 : Details of Mother** | | | | | | | | | | | | | | | | | |
| S.No | Items | | Response | | | | | | | | | | | | | | |
| 1 | Mother’s age in completed years | | years | | | | | | | | | | | | | | |
| 2 | LMP | |  | | | | | | | | | | | | | | |
| 3 | EDD | |  | | | | | | | | | | | | | | |
| 4 | Delivery Details | Normal Delivery | Spontaneous/Induced | | | | | | | | | | | | | | |
|  |  | Instrumental | Forceps/Vacuum | | | | | | | | | | | | | | |
|  |  | LSCS | Elective/Emergency  Indication____________________ | | | | | | | | | | | | | | |
| 5 | Antenatal Steroids | | Y / N  If Yes complete/Partial/Multiple course | | | | | | | | | | | | | | |
| 6 | APGAR score at 1/5/10 min | | / / | | | | | | | | | | | | | | |
| 7 | Intrauterine growth status | | SGA/AGA/LGA | | | | | | | | | | | | | | |
| 8 | Address | | **House number** | | | | | | | | | | | | | | |
|  |  |  | **Street number/ Street name** | | | | | | | | | | | | | | |
|  |  |  | **Locality / VPO / District** | | | | | | | | | | | | | | |
|  |  |  | **City / State** | | | | | | | | | | | | | | |
|  |  |  | PIN CODE |  | |  | | |  | |  | |  | | |  | |
|  |  |  |  |  |  |  |  |  |  |  |  |  |  |  |  |  |  |
|  |  |  | Mobile 1 |  |  | |  |  | |  |  |  | |  |  | |  |
|  |  |  | Mobile 2 |  |  | |  |  | |  |  |  | |  |  | |  |
| **Part A-2: Nutrition** | | | | | | | | | | | | | | | | | |
| S.No | Items | | Response | | | | | | | | | | | | | | |
| 1 | Feeding | | EBM /Partial Breast Feeding/Formula | | | | | | | | | | | | | | |
| 2 | Day of life at which baby is on full feeds | | _______ days | | | | | | | | | | | | | | |
| 3 | Human Milk Fortifiers used | | Y / N Duration _____days | | | | | | | | | | | | | | |
| 4 | IV Fluids | | Y / N Duration _____days | | | | | | | | | | | | | | |
| 5 | Parenteral Nutrition | | Partial/TPN /Not Given Duration of TPN _____days | | | | | | | | | | | | | | |
|  |  | |  | | | | | | | | | | | | | | |
| **Morbidities** | | | | | | | | | | | | | | | | | |
| S.No | Items | | Response | | | | | | | | | | | | | | |
| 1 | Blood culture proven Sepsis | | Y / N Organism_______________ | | | | | | | | | | | | | | |
| 2 | Duration of Antibiotics | | __________________________days | | | | | | | | | | | | | | |
| 3 | Respiratory distress syndrome | | Y / N | | | | | | | | | | | | | | |
| 4 | Study start age (hours) | |  | | | | | | | | | | | | | | |
| 5 | Surfactant | | Y / N | | | | | | | | | | | | | | |
| 6 | Age of giving surfactant (hours) | |  | | | | | | | | | | | | | | |
| 7 | Second dose of surfactant | | Y / N | | | | | | | | | | | | | | |
| 8 | SAS score at time of randomization | |  | | | | | | | | | | | | | | |
| 9 | FiO2 at start of intervention | |  | | | | | | | | | | | | | | |
| 10 | Maximum FiO2 required | |  | | | | | | | | | | | | | | |
| 11 | Flow at start of study | |  | | | | | | | | | | | | | | |
| 12 | Initial choice of interface | |  | | | | | | | | | | | | | | |
| 13 | Age at change of first choice interface (hours) | |  | | | | | | | | | | | | | | |
| 14 | Duration of CPAP ( hours) | |  | | | | | | | | | | | | | | |
| 15 | Duration of Mask as interface ( hours) | |  | | | | | | | | | | | | | | |
| 16 | Duration of Prongs as interface ( hours) | |  | | | | | | | | | | | | | | |
| 17 | Duration of oxygen all forms ( days) | |  | | | | | | | | | | | | | | |
| 18 | Any adverse event during CPAP | | Y / N | | | | | | | | | | | | | | |
| 19 | Maximum nasal injury score | |  | | | | | | | | | | | | | | |
| 20 | Nasal injury score at discharge | |  | | | | | | | | | | | | | | |
| 21 | Pneumonia | |  | | | | | | | | | | | | | | |
| 22 | NEC | | Y / N Stage: _______ | | | | | | | | | | | | | | |
| 23 | PDA | | Y / N Medical / surgical | | | | | | | | | | | | | | |
| 24 | IVH ( grade 3 or 4) | | Y / N Grading________ | | | | | | | | | | | | | | |
| 25 | Cystic PVL (Grade≥2) | | Y / N Grading________ | | | | | | | | | | | | | | |
| 26 | ROP | | Y / N Grading________ | | | | | | | | | | | | | | |
| 27 | ROP requiring laser | | Y / N | | | | | | | | | | | | | | |
| 28 | Supplemental oxygen at 28 days of life, | | Y / N | | | | | | | | | | | | | | |
| 29 | BPD (use of supplemental oxygen at 36 weeks postmenstrual age) | | Y / N | | | | | | | | | | | | | | |
| 30 | Pulmonary air leaks (pneumothorax, PIE) | | Y / N | | | | | | | | | | | | | | |
| 31 | Neonatal mortality | | Y / N | | | | | | | | | | | | | | |
| 32 | Age at discharge (day of life) | |  | | | | | | | | | | | | | | |
| 33 | Weight at discharge (gram) | |  | | | | | | | | | | | | | | |
| 34 | Length at discharge (cm) | |  | | | | | | | | | | | | | | |
| 35 | OFC at discharge (cm) | |  | | | | | | | | | | | | | | |
| 36 | Nasal injury at discharge  Total score:  Grade of injury | |  | | | | | | | | | | | | | | |

**Monitoring chart for nasal injury**

| **Nasal injury** | **Score** | | | | | | | | | | |
| --- | --- | --- | --- | --- | --- | --- | --- | --- | --- | --- | --- |
|  | **Day 1** | **Day 2** | **Day 3** | **Day 4** | **Day 5** | **Day 6** | **Day 7** | **Day 14** | **Day 21** | **Day 28** | **Discharge** |
| **Tip of Nose** |  |  |  |  |  |  |  |  |  |  |  |
|  |  |  |  |  |  |  |  |  |  |  |  |
| **Nasal Septum** |  |  |  |  |  |  |  |  |  |  |  |
|  |  |  |  |  |  |  |  |  |  |  |  |
| **Nostrils** |  |  |  |  |  |  |  |  |  |  |  |
|  |  |  |  |  |  |  |  |  |  |  |  |
| **Nose Shape** |  |  |  |  |  |  |  |  |  |  |  |
|  |  |  |  |  |  |  |  |  |  |  |  |
| **Bridge of the nose** |  |  |  |  |  |  |  |  |  |  |  |
| **Upper lip** |  |  |  |  |  |  |  |  |  |  |  |
| **Total score** |  |  |  |  |  |  |  |  |  |  |  |

| PATIENT END | | | | | MACHINE END | | | | | Interface and settings | | |
| --- | --- | --- | --- | --- | --- | --- | --- | --- | --- | --- | --- | --- |
|  | Distance between cannula& interface-2mm | Nasal skin blanched | Columella/ septal excoriation | Oral/nasal suction done | Bubble present | Temperature normal | Tubings below patient level | Flow rate  5-8 L/min | Water level (Humidifier& Container) | Mask or prong | FiO2 | PEEP |
| 0800 |  |  |  |  |  |  |  |  |  |  |  |  |
| 1200 |  |  |  |  |  |  |  |  |  |  |  |  |
| 1600 |  |  |  |  |  |  |  |  |  |  |  |  |
| 2000 |  |  |  |  |  |  |  |  |  |  |  |  |
| 0000 |  |  |  |  |  |  |  |  |  |  |  |  |
| 0400 |  |  |  |  |  |  |  |  |  |  |  |  |
| Signature |  |  |  |  |  |  |  |  |  |  |  |  |

CPAP Monitoring chart

Name of the baby: MR No: Enrolment No: Group allocated:

**ANNEXURE - 2**

**PATIENT INFORMATION SHEET**

**TITLE** : Can rotation of nasal interfaces decrease the incidence of nasal injury in neonates on nasal CPAP? – A Randomized control trial

Principal investigator: Dr Tanveer Bashir

Chief Guide: Dr Srinivas Murki

Dear Parent,

You are invited to participate in the above mentioned study. You have been asked to consider whether or not you wish to enter your baby for the study. You are entirely free to decide whether or not you want to enter your baby into this study. Whatever may be your decision your baby will receive the best possible treatment at the hospital.

**What is the purpose of the study?**

Premature babies are at high risk of respiratory distress and they need respiratory support which should be started early and should be optimal. Inserting an endotracheal tube and giving intermittent positive pressure breaths is the conventional mode of ventilation in these babies. Though it is effective, it is associated with various complications. Hence, various non-invasive modalities like CPAP are used. CPAP (continues positive airway pressure) device is one of the non-invasive mode of ventilation which will help in stabilising the respiratory status of the neonate by opening alveoli (terminal ends of the respiratory tree). It is well proven that early use of CPAP, will reduce the need for subsequent intubations. Nasal mask and nasal prong are commonly used as interfaces to deliver CPAP. Both nasal interfaces are associated with some form of nasal injuries (injury to nostrils in the form of redness, excoriation or deformity to the bridge of the nose). In this study we will see whether rotation of nasal interfaces will reduce incidence of nasal injury compared to individual interfaces.

**What will happen if you give consent ?**

If your baby developed respiratory difficulty and requires CPAP as per the existing guidelines will be considered eligible for the study. Your baby will be put on CPAP with nasal prong or nasal mask as interface and will receive CPAP according to unit protocol.

**What are the benefits to your baby?**

Though there are no direct benefits to your baby, the result of the study will guide to decide regarding the better CPAP delivery interface device. This study may also benefit similar babies at large in future.

**What are the risks to your baby?**

No significant extra blood test or any other test is required for the study and both interfaces are equally effective to deliver CPAP. So there is no risk to your baby.

**Maintenance of confidentiality of records**

All the information that you provide during the study will be kept confidential.

**How will your decision to not participate in the study affect you?**

Your decision not to participate in this research study will not affect your medical care or your relationship with the investigator or the institution. Your doctor will still take care of your baby as he would have done in any case without compromising the quality of care and you will not loose any benefits to which you are entitled.

**Can you decide to stop participating in the study once you start?**

Participation in this research is purely voluntary and you have the right to withdraw from this study at any time during the course of the study without giving any reasons. Though advisable that you give the investigators the reason for withdrawing, it is not mandatory.

**Right to new information**

If the research team gets any new information during this research study that may affect your decision to continue participating in the study, or may raise some doubts, you will be told about that information.

You have the right to refuse the individual procedures. If you have any further questions, please contact Dr Tanveer, DNB Fellow in Neonatology, Department of Neonatology.

**Contacts:** In the event that at any time during the course of the study you feel that you have not been adequately informed as to the risks, benefits, alternative procedures, or your rights as a study subject or feel under duress to continue against your wishes you can contact:

Co-Investigators

Dr. Srinivas murki, Consultant Neonatology, Department of Neonatology, Fernandez Hospital, Hyderguda, Hyderabad. Ph no: 9392470351

Dr. Tejopratap Oleti, Consultant Neonatology, Department of Neonatology, Fernandez Hospital, Hyderguda, Hyderabad. Ph no: 9989372490

**ANNEXURE - 3**

**INFORMED CONSENT FORM**

Subject identification number for this trail

Title of the project_ Can rotation of nasal interfaces decrease the incidence of nasal injury in neonates on nasal CPAP? – A Randomized control trial

Name of the principal investigator Dr. Tanveer Bashir. Tel No.9515364962

I have received the information sheet on the above study and have read and /or understood the written information.

I have been given the chance to discuss the study and ask questions.

I consent to take part in the study and I am aware that my participation is voluntary

I understand that I may withdraw at any time without this affecting my future care.

I understand that the information collected about me from my participation in this research and sections of any of my medical notes may be looked at by responsible persons(ethics committee members/regulatory authorities).I give access to these individuals to have access to my records.

I understand I will receive a copy of the patient information sheet and the informed consent form.

_______________________ ______________

Signature/Thumb Impression of Subject Date of Signature

____________________________

Printed name of the subject in capitals

______________________________ ___________________

Signature /Thumb Impression of legally Date of Signature

Accepted representative

<< The legally acceptable representitives signature should be added if the subject is a minor or is unable to sign for themselves.The relationship between the subject and the legally acceptable representative should be stated. The impartial witness signature should be added if the subject/legally acceptable representitives is unable to read or write and consent should be obtained in his presence.>>

_________________________________________________

Printed name of legally acceptable representative in capitals.

__________________________________________________

Relationship of legally accepted representative to subject in capitals.

_________________________ _________________

Signature of the person conducting the Date of signature informed consent discussion

_______________________________

Printed name of the person conducting the informed consent discussion in capitals.

___________________________ ______________

Signature of impartial witness Date of signature

____________________________

Printed name of the impartial witness in capitals
